# Keeping the network alive: The importance of professional social networks for long-term research career development

**DOI:** 10.1017/cts.2025.10105

**Published:** 2025-08-15

**Authors:** Marisa N. Spann, May Hua, Daichi Shimbo, Melissa Begg, Robyn Gartrell, Fatemeh Momen-Heravi, Irving Institute for Clinical Translational Research, Melissa Accordino, Eileen Connolly, Andrew Einstein, Katherine Dimitropoulou, Bernard Chang, Badri Vardarajan

**Affiliations:** 1 Vagelos College of Physicians and Surgeons, Columbia University, New York, NY, USA; 2 Columbia School of Social Work, New York, NY, USA; 3 Johns Hopkins University School of Medicine, Baltimore, MD, USA; 4 School of Dentistry, University of California, San Francisco, USA

**Keywords:** Alumni, career development, networking, mentoring, Kl2

Professional networking involves building intentional relationships to advance career development [1,2]. Networks facilitate resource exchange, collaboration, skill development, and career progression [3–5]. Networks transfer tacit knowledge, spur innovation, and promote collaboration, mentorship, and resource access [6,7]. They also amplify social capital [8,9], visibility, and leadership opportunities [10–13]. While extremely valuable, networks can be challenging for an individual to establish or join if they are already in existence [4,14]. Thus, infrastructural support for professional networks beyond formal training programs could be advantageous. Therefore, we propose the need for infrastructural support for sustained and effective networking communities for multistage faculty at institutions, as well as models and guidelines of how to build them so they can be successful in enhancing career resilience and long-term success.

Past studies have underscored networks’ role in academic achievement. For example, Robinson et al. (2016) found that early-career awardees (NIH KL2/K12; *N* = 40) credited institutional, national, and international networks as key to their success [15]. Viglianti et al. 2022, present a peer mentorship network of early career clinician-scientists in academic medicine (Multidisciplinary Intensive Care Research Workgroup-MICReW) [16]. The program fostered accountability, exchange of opinions and feedback, and encouraged comradery and teamwork. Over the course of seven years, the peer network supported members to progress in academic positions and receive career development awards [16].

Studies highlight the lack of structured professional networks to support scholars facing early and mid-career challenges [17,18]. Llewellyn et al., 2021 present interview and focus group findings from alumni in a structured training seminar for pursuing NIH K awards (K-Club) [17]. Participants expressed the need for additional programs with a more flexible training format and opportunities for small-group interactions that can support professional networks [17]. Professional networking cannot be left to the spontaneous interactions and intuitive social skills of individual scholars. Instead, it is best fostered through infrastructures conducive to this process and is a need that trainees have identified. Martin, et al. 2023, present the notion and need for distributed mentoring in the context of networks formed by scholars from various experience levels. These networks can facilitate interactions and provide feedback, advice, and informal instruction [19]. Despite the need and benefits of structured professional networks, effective models and practices have received minimal scholarly attention [18,20].

## Program Genesis

Columbia University Irving Medical Center’s (CUIMC), Clinical and Translational Science Award (CTSA) program includes TRANSFORM (TRaining And Nurturing Scholars FOr Research that is Multidisciplinary), which is the education core that supports research skill development, pilot funding, and career resources. The KL2 program as well as the TL1 are housed within TRANSFORM. The KL2 provided academic and funding support, essential to early successes as clinician-scientists. The conclusion of the program posed the risk of losing the collaborative environment that had nurtured KL2 scholars’ growth transitioning in 2017. To address this, we designed a program, EVOLUTION (Empowering Voices Of Leaders Using Training, Integration, and Ongoing Networks) that would extend support by building a professional network of alumni. EVOLUTION was designed to support career transitions from K to R/Center-level funding through intentional networking.

TRANSFORM EVOLUTION was born from scholars’ desire to create a structured community. By 2020, its mission expanded to build a sustainable, inclusive network fostering professional collaboration and social connection across career stages. The name “EVOLUTION” reflects its vision: an adaptive community responding to scholars’ evolving needs, emphasizing how professional networks enhance development. In translational science’s demanding landscape, we believe such peer networks will shape the field’s future and empower new generations of researchers.

## Structure and Innovation

TRANSFORM EVOLUTION provides a structure for developing successful intentional professional networks (Table 1). EVOLUTION has been strengthened by the infrastructure provided through the Columbia University Irving Medical Center’s CTSA. The program operates with dedicated financial resources including a small budget for events per year. The support also included 2–4 hours per month of administrative support for coordinating logistics, managing communication, and sustaining engagement across the network. This integrative support structure enhances the program’s capacity for resource sharing, and long-term sustainability. CTSAs are uniquely positioned to offer this type of backbone infrastructure, spanning programmatic coordination, administrative continuity, and cross-campus integration that is critical for building and maintaining successful professional networks.

**Table 1.** TRANSFORM EVOLUTION framework

Characteristics/Structures	Description	Who Participates (Frequency)
Leadership, Infrastructure:	Program Manager & Committee Funded by CTSA Governance Committee	– CUIMC (across 4 schools) – CTSA KL2 trainees, current and alumni – Irving Scholars, current and alumni – Career stage: junior faculty, mid to senior career faculty or non-academic professionals
Communication systems:	Open and transparent information distribution CTSA listservs and email lists	
Community Engagement:		
• In person, or zoom Interactions	Interdisciplinary research forums with interactive discussions/problem-solving	
• Platform for information sharing	Dedicated platform for sharing resources, seek advice, career-focused discussions	
Building Relationships:		
• **Horizontal** (casual connections, build trust & comradery)	Social events (twice/year)	– Same as above
	Pipeline networking of trainees	– Campus-wide TL1 pre- and post-doctoral trainees – CUIMC CTSA KL2 trainees, current – K to R Transition Program
• **Deep relationships** (close systematic connections)	Research groups & mentorship teams	– Governance committee – Individual connections between any faculty or non-academic professionals through network – Current KL2 – Mid-career bubble subgroup
Building Skills:		
• Centralized activities	**Career Development Seminars on topics including grantsmanship, leadership, team science, and work-life balance. Examples provided below:** Fish Bowl Discussion – Problem Solving Challenges in Academia Go for two Golds! Tips and Tricks from Super Grant Awardees Pearls from the New Medical Center Dean Transitioning to Mid-career and Post-tenure: Shifting Mindset and Goals Choosing between K and R Award Mechanisms, or some Combination Tenure or its Equivalent, Setting Yourself on a Path for Job Security and Freedom in Scientific Inquiry Building our Careers: Swimming Forward	– CUIMC (across 4 schools) – CTSA KL2 trainees, current and alumni – Irving Scholars, current and alumni – Career stage: junior faculty, mid to senior career faculty or non-academic professionals
• Decentralized activities	Informal meetings based on topics	– Subgroups interested in specific topics connect

Abbreviations: CTSA = Clinical and Translational Science Award; CUIMC = Columbia University Irving Medical Center; Mentored Research Career Development Program Award, KL2; Ruth L. Kirschstein National Research Service Award (NRSA) Research Training Grant, TL1; Investigator-initiated Research Project (R01).

TRANSFORM EVOLUTION represents a novel approach to professional networking by creating an enduring, cross-career community for program alumni. Distinct from traditional hierarchical models that separate senior and junior scholars or peer networks limited to similar career stages, EVOLUTION intentionally integrates researchers across all academic ranks into a flexible, needs-responsive ecosystem. This innovative structure fosters horizontal collaboration where participants collectively problem-solve, generate ideas, develop new methodologies, and exchange insights regardless of career stage. By transcending conventional mentorship paradigms, the program addresses a critical gap in the literature while creating dynamic synergies that benefit early-career trainees and established faculty alike through participation and mutual support.

## Impact and Continuity

**Figure 1.**
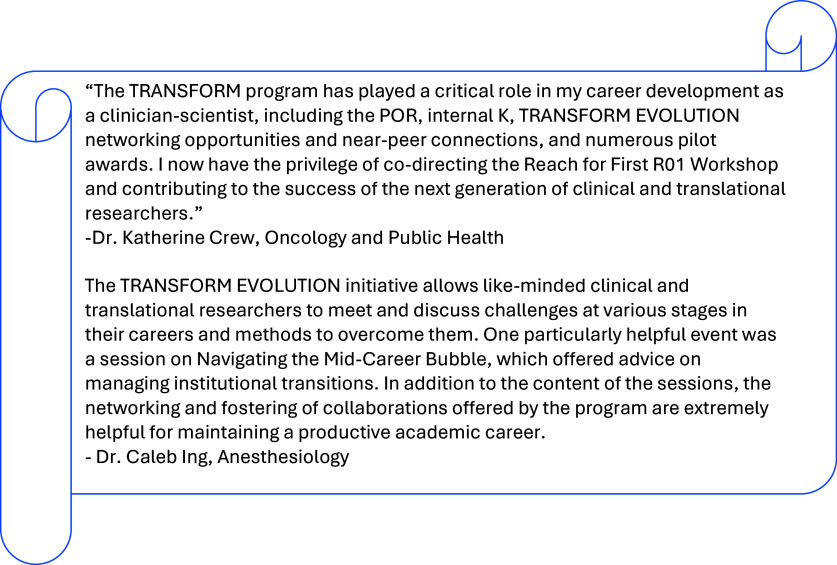
Testimonials from TRANSFORM EVOLUTION network members about their experiences with the programming. Abbreviations: TRANSFORM = TRaining and nurturing scholars FOr research that is multidisciplinary; EVOLUTION = empowering voices of leaders using training, integration, and ongoing networks; mentored research career development program award KL2, internal K; POR = patient-oriented research colloquium.

The TRANSFORM EVOLUTION network fosters a sense of personal connection and belonging, giving members access to peer scholars and opportunities for collaboration and mentorship. It has positively impacted careers of many researchers (Figure 1).

At the core of TRANSFORM EVOLUTION is a deep commitment to the principles of mentorship and the importance of professional social networks. The program’s founders believed that an effective community would need to include multistage faculty to ensure peer and near peer support. Networking events are well-attended by faculty at every career stage. For example, events occurring between April 2022 and December 2024 had 42 unique attendees from every career stage, with some attendees having had no NIH grants and others having had 14 NIH grants in their academic lifetime. While senior faculty provide invaluable guidance based on their extensive experience, junior faculty contribute fresh perspectives and innovative approaches having recently navigated some of the benchmarks, applications, and promotion processes enriching the development of a support network. Exposure to structured leadership training and real-world mentorship experiences has equipped early career scholars with the skills needed to transition into independent investigator roles. The program’s robust KL2 alumni network plays a critical role in maintaining this culture. Alumni, many of whom are now leaders in their respective fields, remain actively engaged in the program and attend the networking events. This engagement not only provides current participants with access to seasoned colleagues but also allows alumni to contribute to the ongoing development of the next generation of clinician-scientists.

## Sustainability and Future Directions

From its inception, TRANSFORM EVOLUTION was designed with sustainability in mind. A governance committee composed of scholars at different stages provides leadership, ensuring that programming remains responsive and relevant. We are planning to further define effective practices and structures of the program and establish program outcomes to monitor its success. Looking ahead, the program aims to expand cross-institutional collaborations with other CTSA sites and translational research networks while also developing robust evaluation frameworks to track participants’ progress and refine offerings. In the long term, we aim to disseminate our program regionally and nationally, integrating it into the broader academic and professional development infrastructure, through our career development sessions that could include smaller group break outs. In translational science’s demanding landscape, we believe such peer networks will shape the field’s future and empower new generations of researchers.

## References

[1] Dunn MB. Early career developmental networks and professionals’ knowledge creation. J Manag. 2019;45:1343–1371. doi: 10.1177/0149206317702218.

[2] Selvarajah K , Zadeh PM , Kobti Z , Palanichamy Y , Kargar M. A unified framework for effective team formation in social networks. Expert Syst Appl. 2021;177:114886. doi: 10.1016/j.eswa.2021.114886.

[3] Gibson C , Hardy JH III , Buckley MR. Understanding the role of networking in organizations. Career Dev Int. 2014;19:146–161. doi: 10.1108/CDI-09-2013-0111.

[4] Porter CM , Woo SE , Alonso N , Snyder G. Why do people network? Professional networking motives and their implications for networking behaviors and career success. J Vocat Behav. 2023;142:103856. doi: 10.1016/j.jvb.2023.103856.

[5] Fischer LF. Sharing is caring? Knowledge diffusion in researcher networks. SSRN Electron J. 2024. doi: 10.2139/ssrn.4262428.

[6] Heffernan T. Academic networks and career trajectory: “There’s no career in academia without networks. High Educ Res Dev. 2021;40:981–994. doi: 10.1080/07294360.2020.1799948.

[7] Ansmann L , Flickinger TE , Barello S , et al. Career development for early career academics: benefits of networking and the role of professional societies. Patient Educ Couns. 2014;97:132–134. doi: 10.1016/j.pec.2014.06.013. 25074842

[8] Burt RS. Brokerage and closure: An introduction to social capital. Oxford University Press, 2005.

[9] Mehra A , Dixon AL , Brass DJ , Robertson B. The social network ties of group leaders: implications for group performance and leader reputation. Organ Sci. 2006;17:64–79. doi: 10.1287/orsc.1050.0158.

[10] Cola PA , Wang Y. Discovering factors that influence physician scientist success in academic medical centers. Qual Health Res. 2022;32:1433–1446. doi: 10.1177/10497323221108639. 35737579

[11] Goel RK , Grimpe C. Active versus passive academic networking: evidence from micro-level data. J Technol Transf. 2013;38:116–134. doi: 10.1007/s10961-011-9236-5.

[12] Storme T , Faulconbridge JR , Beaverstock JV , Derudder B , Witlox F. Mobility and professional networks in academia: an exploration of the obligations of presence. Mobilities. 2017;12:405–424.

[13] Rubio DM , Primack BA , Switzer GE , Bryce CL , Seltzer DL , Kapoor WN. A comprehensive career-success model for physician–scientists. Acad Med. 2011;86:1571–1576. doi: 10.1097/ACM.0b013e31823592fd. 22030759 PMC3228877

[14] Goolsby MJ , Knestrick JM. Effective professional networking. J Am Assoc Nurse Pract. 2017;29:441–445. doi: 10.1002/2327-6924.12484.7. 28608520

[15] Robinson GF , Schwartz LS , DiMeglio LA , Ahluwalia JS , Gabrilove JL. Understanding career success and its contributing factors for clinical and translational investigators. Acad Med. 2016;91:570–582. doi: 10.1097/ACM.0000000000000979.8. 26509600 PMC4811729

[16] Viglianti EM , Admon AJ , Carlton EF , et al. Development and retention of early-career clinician–scientists through a novel peer mentorship program: multidisciplinary intensive care research workgroup. ATS Scholar. 2022;3:588–597. doi: 10.34197/ats-scholar.2022-0039IN. 36726705 PMC9886001

[17] Llewellyn NM , Adachi JJ , Nehl EJ , Heilman SS. Participant perspectives on a seminar-based research career development program and its role in career independence. J Investig Med. 2021;69:775–780. doi: 10.1136/jim-2020-001769. PMC8083062 33602694

[18] Clegg S , Josserand E , Mehra A , Pitsis TS. The transformative power of network dynamics: a research agenda. Organ Stud. 2016;37:277–291. doi: 10.1177/01708406166290.

[19] Martin A , Mori J , Froehlich DE. Career development of early career researchers via distributed peer mentoring networks. Merits. 2023;3:569–582. doi: 10.3390/merits3030034.

[20] Bian J , Xie M , Topaloglu U , Hudson T , Eswaran H , Hogan W. Social network analysis of biomedical research collaboration networks in a CTSA institution. J Biomed Inform. 2014;52:130–140. doi: 10.1016/j.jbi.2014.01.015. 24560679 PMC4136998

